# Long COVID and gut microbiome: insights into pathogenesis and therapeutics

**DOI:** 10.1080/19490976.2025.2457495

**Published:** 2025-01-24

**Authors:** Raphaela I. Lau, Qi Su, Siew C. Ng

**Affiliations:** a Department of Medicine and Therapeutics, Faculty of Medicine, The Chinese University of Hong Kong, Hong Kong SAR, China; b Microbiota I-Center (MagIC), Hong Kong SAR, China; c Li Ka Shing Institute of Health Sciences, State Key Laboratory of Digestive Disease, Institute of Digestive Disease, The Chinese University of Hong Kong, Hong Kong SAR, China

**Keywords:** Long COVID, post-acute COVID-19 syndrome, gut microbiome, microbiome-based therapeutics

## Abstract

Post-acute coronavirus disease 2019 syndrome (PACS), following severe acute respiratory syndrome coronavirus 2 (SARS-CoV-2) infection or coronavirus disease 2019 (COVID-19), is typically characterized by long-term debilitating symptoms affecting multiple organs and systems. Unfortunately, there is currently a lack of effective treatment strategies. Altered gut microbiome has been proposed as one of the plausible mechanisms involved in the pathogenesis of PACS; extensive studies have emerged to bridge the gap between the persistent symptoms and the dysbiosis of gut microbiome. Recent clinical trials have indicated that gut microbiome modulation using probiotics, prebiotics, and fecal microbiota transplantation (FMT) led to improvements in multiple symptoms related to PACS, including fatigue, memory loss, difficulty in concentration, gastrointestinal upset, and disturbances in sleep and mood. In this review, we highlight the latest evidence on the key microbial alterations observed in PACS, as well as the use of microbiome-based therapeutics in managing PACS symptoms. These novel findings altogether shed light on the treatment of PACS and other chronic conditions.

## Introduction

Post-acute coronavirus disease 2019 syndrome (PACS) is a multisystemic condition characterized by a range of debilitating symptoms that persist following severe acute respiratory syndrome coronavirus 2 (SARS-CoV-2) infection or coronavirus disease 2019 (COVID-19).^1^ The cumulative global incidence of PACS is estimated to be over 409 million since 2020.^2^ Although SARS-CoV-2 primarily affects the respiratory tract, PACS could impact multiple organs and systems, resulting in a range of persistent symptoms. These may include respiratory symptoms (such as coughing and dyspnea), cardiovascular symptoms (such as chest pain and palpitations), gastrointestinal symptoms (such as diarrhea and constipation), psychiatric symptoms (such as anxiety and sleep disturbances), cognitive symptoms (such as memory impairment and difficulty concentrating), musculoskeletal symptoms (such as joint and muscle pain), dermatological symptoms (such as rashes and alopecia), and other manifestations (such as fatigue and malaise).^1,3–6^ Individuals with PACS may experience chronic health conditions, leading to a decline in quality of life and physical functioning.^4,7^ Furthermore, PACS poses a detrimental impact on various aspects of a patient’s daily activities, including work, study, and interpersonal relationships.^8–10^

The exact pathogenesis of PACS remains uncertain. However, several mechanisms that may contribute to the development of PACS have been hypothesized, including the persistence of viral ribonucleic acid (RNA) and proteins, infection-induced autoimmune dysregulation, latent viral reactivation, inflammation-induced tissue dysfunction and damage, as well as gut microbiome dysbiosis.^11–13^ The gut microbiome is a diverse community of microorganisms (i.e., bacteria, viruses, fungi, archaea) that reside along the gastrointestinal tract and play roles in regulating host immunity, defending against pathogens, and supporting nutrient metabolism.^14^ Gut microbiome dysbiosis with a reduction in microbial diversity is linked to acute COVID-19, potentially through the bi-directional crosstalk between the gut microbiome and the respiratory system, commonly known as the gut-lung axis.^15^ Extensive studies have emerged to bridge the gap between the persistent symptoms and the dysbiosis of the gut microbiome. Hence, the gut microbiome could be a promising therapeutic target for PACS.

Here, we examine the latest evidence on the key microbial alterations observed in PACS, and the use of microbiome-based therapeutics in alleviating PACS symptoms. Furthermore, we discuss future research directions to translate these microbiome discoveries into clinical applications beyond the pandemic.

## Methods

We have reviewed observational studies, animal studies, clinical trials, case reports, systematic reviews, and meta-analyses on PubMed/MEDLINE and Google Scholar published between December 2019 and December 2024 on the key microbial alterations observed in PACS, as well as the potential efficacy of gut microbiome-based therapeutics in the treatment of PACS, using keywords including “COVID-19” or “post-COVID” or “post-acute COVID-19 syndrome” or “long COVID” or “PACS” or “PASC” or “PCC”, combined with “microbiome” or “microbiota” or “mycobiota” or “probiotic” or “prebiotic” or “synbiotic” or “postbiotic” or “parabiotic” or “paraprobiotic” or “FMT” or “fecal microbiota transplantation” or “antibiotic” or “diet” or “nutrition”.

## Role of gut microbiome and key microbial alterations in PACS

To date, a number of observational studies have reported alterations in gut microbiome composition in PACS patients.^5,16–28^ These studies were conducted in different countries and regions, including China, Brazil, Hong Kong, Japan, Latvia, Norway, Russia, and the United States, with details summarized in Table 1.

**Table 1.** Overview of key microbial alterations in post-acute coronavirus disease 2019 syndrome (PACS).

Location	Study participants	Timepoint	Key microbial changes	Reference
China	15 COVID-19 patients; 14 healthy controls	3 months after discharge	Reduced bacterial diversity in recovered COVID-19 patients Positively correlated with PACS: *Escherichia* unclassified, *Intestinibacter bartlettii* Negatively correlated with PACS: *Faecalibacterium prausnitzii*, *Intestinimonas butyriciproducens*	Zhou^16^
China	45 COVID-19 patients; 25 healthy controls	3 months after discharge	Depleted bacterial genera in gastrointestinal PACS: *Prevotella*, *Neisseria*, *Streptococcus*, *Haemophilus*, *Veillonella*, *Alloprevotella*, *Porphyromonas*, *Actinomyces*, *Fusobacterium*, *Rothia*	Zhang^17^
China	30 COVID-19 patients; 30 healthy controls	6 months after discharge	Reduced microbial richness in recovered COVID-19 patients	Chen^18^
China	35 COVID-19 patients; 160 healthy controls	at discharge; 1 year after discharge	Microbial diversity, butyric acidproducing microbes and *Bifidobacterium* gradually increased; lipopolysaccharideproducing microbes gradually decreased at 1 year after discharge	Cui^19^
China	130 COVID-19 patients, 32 healthy controls	1 year after discharge	Reduced bacterial diversity in PACS Enriched bacterial genera in patients with PACS: *Veillonella*, *Erysipelatoclostridium* Depleted bacterial genera in patients with PACS: *Eubacterium hallii* group, *Agathobacter*, *Subdoligranulum*, *Ruminococcus*, *Dorea*, *Coprococcus*, *Eubacterium ventriosum* group	Zhang^20^
Brazil	149 COVID-19 patients; 71 healthy controls	1–8 months after acute COVID-19	Enriched bacterial genera in patients: *Parabacteroides*, *Bacteroides*, *Alistipes*, *Dynosmobacter*, *Butyricimonas*, *Bilophila*, *Flavonifractor*, *Barnesiella*, *Anaerotignum*, *Parasutterella*, *Acidaminococcus* Depleted bacterial genera in patients: *Dorea*, *Streptococcus*, *Bifidobacterium*, *Akkermansia*	Ferreira-Junior^21^
Hong Kong, China	68 COVID-19 patients; 68 healthy controls	6 months after discharge	Reduced bacterial diversity and richness in PACS Enriched bacterial species in PACS: *Ruminococcus gnavus*, *Bacteroides vulgatus* Depleted bacterial species in PACS: *Faecalibacterium prausnitzii*	Liu^5^
Hong Kong, China	155 COVID-19 patients; 155 healthy controls	14 months after viral clearance	Reduced bacterial diversity and richness in PACS Enriched bacterial species in PACS: *Ruminococcus gnavus*, *Clostridium bolteae*, *Flavonifractor plautii*, *Erysipelatoclostridium ramosum* Depleted bacterial species in PACS: *Gemmiger formicilis*, *Bifidobacterium adolescentis*, *Bifidobacterium pseudocatenulatum*	Su^22^
Japan	2 PACS patients living with human immune-deficiency virus	128 and 382 days after COVID-19 onset	Enriched bacterial taxa: *Enterobacteriaceae*, *Escherichia-Shigella*, *Phascolarctobacterium*, *Megamonas* Depleted bacterial taxa: *Bifidobacterium*	Ishizaka^23^
Latvia	78 COVID-19 patients with PACS; 44 COVID-19 patients without PACS	20 days after COVID-19 onset	Reduced bacterial diversity in patients later experiencing PACS Enriched bacterial genus in patients later experiencing PACS: *Prevotella*	Brīvība^24^
Norway	83 COVID-19 patients	3 months after admission	Reduced bacterial diversity in patients with respiratory dysfunction Enriched bacterial genera in patients with respiratory dysfunction: *Flavonifractor*, *Veillonella* Depleted bacterial genera in patients with respiratory dysfunction: *Erysipelotrichaceae* UCG-003 and several members of the *Lachnospiraceae* and *Ruminococcaceae* family	Vestad^25^
Russia	39 PACS patients; 48 healthy controls	More than 3 months after acute COVID-19	Elevated total bacterial mass and *Bacteroides* species in most PACS patients	Sorokina^26^
Russia	134 COVID-19 patients; 46 healthy controls	3 months after recovery	Positively associated with endothelial dysfunction markers: *Romboustia*, *Ruminococcus gnavus* Negatively associated with endothelial dysfunction markers: *Lachnospiraceae* groups UCG-010 and NK4A136, *Barnesiella*, *Eubacterium xylanophillum*	Tkacheva^27^
United States	19 COVID-19 patients with PACS; 15 COVID-19 patients without PACS	More than 4 weeks after COVID-19 onset	Lowered ratio of an amplicon sequence variant (ASV) associated with *Faecalibacterium prausnitzii* over ASVs associated with *Bacteroides* in patients with PACS	Carneiro^28^

COVID-19, coronavirus disease 2019; PACS, post-acute COVID-19 syndrome.

**Figure 1.**
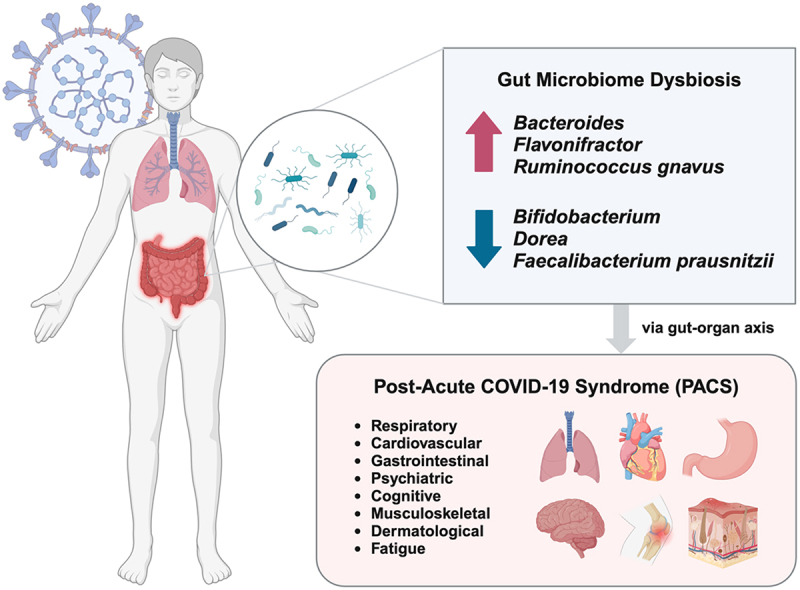
Gut microbiome dysbiosis in post-acute coronavirus disease 2019 syndrome (PACS). In PACS patients across existing published studies, gut bacterial genera that have consistently shown positive association with PACS across studies include *Bacteroides* ^21,26,28^ and *Flavonifractor*,^21,25^ while those with a consistently negative association include *Bifidobacterium* ^21,23^ and *Dorea*.^20,21^ On the species level, *Ruminococcus gnavus* has consistently shown positive association with PACS across studies,^5,22,27^ whilst *Faecalibacterium prausnitzii* has consistently shown negative association.^5,16,28^ These microbial signatures were found to be associated with long-term debilitating symptoms affecting multiple organs and systems, including respiratory symptoms (e.g., coughing and dyspnea), cardiovascular symptoms (e.g., chest pain and palpitations), gastrointestinal symptoms (e.g., diarrhea and constipation), psychiatric symptoms (e.g., anxiety and sleep disturbances), cognitive symptoms (e.g., memory impairment and difficulty concentrating), musculoskeletal symptoms (e.g., joint and muscle pain), dermatological symptoms (e.g., rashes and alopecia), and other manifestations (e.g., fatigue and malaise). COVID-19, coronavirus disease 2019; PACS, post-acute COVID-19 syndrome. Figure created with BioRender.com.

Compared to healthy controls, the gut microbiome of PACS patients was characterized by gut microbiome dysbiosis with reduced microbial diversity and richness.^5,16,18,20,22,24,25^ As shown in Figure 1, bacterial genera that have consistently shown positive association with PACS across studies include *Bacteroides* ^21,26,28^ and *Flavonifractor*,^21,25^ while those with a consistently negative association include *Bifidobacterium* ^21,23^ and *Dorea*.^20,21^ Contradictory findings were observed for the *Barnesiella*,^21,27^ *Prevotella*,^17,24^ *Streptococcus* ^17,21^ and *Veillonella* ^17,20,25^ genera. On the species level, *Ruminococcus gnavus* has consistently shown positive association with PACS across studies,^5,22,27^ whilst *Faecalibacterium prausnitzii* has consistently shown negative association.^5,16,28^ In fact, an elevation of *Ruminococcus gnavus* and a depletion of *Faecalibacterium prausnitzii* are both linked to multiple diseases of different organs and systems, including inflammatory bowel disease (IBD), irritable bowel syndrome (IBS), colorectal cancer, obesity, type 2 diabetes, psychiatric disorders, and neurodegenerative disorders,^29,30^ resembling the multi-systemic nature of PACS. Beyond bacterial dysbiosis, several studies have also provided insights on the associations between fungal dysbiosis, severity of acute COVID-19, and PACS.^31–34^ In particular, an increase in opportunistic fungal pathogens including *Candida* have been observed in patients with severe COVID-19,^31,32,34^ and evidence is emerging in regard to the possible relationship between gut candidiasis and PACS.^35^ Nonetheless, all of the above bacterial and fungal changes related to COVID-19 and PACS were highly heterogeneous across studies, which may be due to the influence of dietary, lifestyle, environmental, and genetic factors on gut microbiome composition, considering that many of the fecal samples were collected months after symptom onset. Therefore, personalized interventions targeting different microbial compositions might be crucial.

Only a few studies have investigated into relationships between specific symptoms of PACS and key changes in the gut microbiome. A study in China has collected fecal samples from patients with gastrointestinal PACS symptoms 3 months after hospital discharge and has detected depletion of several bacterial genera in the gut, including *Prevotella*, *Neisseria*, *Streptococcus*, *Haemophilus*, *Veillonella*, *Alloprevotella*, *Porphyromonas*, *Actinomyces*, *Fusobacterium* and *Rothia*.^17^ In a study conducted in Norway, PACS patients with respiratory dysfunction 3 months after hospital admission have exhibited an enrichment of *Flavonifractor* and *Veillonella*, along with a depletion of *Erysipelotrichaceae* UCG-003 and several members of the *Lachnospiraceae* and *Ruminococcaceae* families in the gut.^25^ Another study in Hong Kong has explored the longitudinal alterations of the gut microbiome in hospitalized COVID-19 patients within 6 months after SARS-CoV-2 infection.^5^ It was found that respiratory PACS symptoms were linked to heightened levels of opportunistic pathogens such as *Streptococcus vestibularis* and *Streptococcus anginosus*. Fatigue and neuropsychiatric symptoms, on the other hand, were linked to bacteria such as *Clostridium innocuum* and *Actinomyces naeslundii*. Gut bacteria that are known to produce butyrate were also significantly depleted in patients with hair loss, with certain bacteria including *Bifidobacterium pseudocatenulatum* and *F. prausnitzii* showing the greatest inverse correlations with PACS. Last but not least, a study has identified associations between gut microbiome-based enterotypes and particular PACS symptoms.^36^ Three enterotypes with distinct microbial composition and functions were independently linked to respiratory symptoms, neurological symptoms, or fatigue. The utilization of a multi-label machine learning model developed using gut microbiome data was demonstrated to achieve accurate prediction of individual PACS symptoms. Collectively, these findings reflect the potential contribution of the gut microbiome in individual symptoms of PACS. Further studies would be important to reveal the key microbial changes associated with individual symptoms across PACS populations.

Furthermore, the gut microbiome may mediate the effects of host factors on the development of PACS. Female sex, older age, more severe acute COVID-19, higher body mass index (BMI), as well as the presence of comorbidities were commonly reported as potential risk factors of PACS across a number of retrospective, prospective, and cross-sectional studies.^37–41^ A systematic review and meta-analysis of 41 articles reporting on 860,783 subjects has also revealed that female sex, older age, higher BMI, smoking, comorbidities, previous hospital, or intensive care unit stay were related to heightened risk of PACS.^42^ Subjects who received two doses of COVID-19 vaccination were also found to be related to lowered risk of PACS when compared to those who were not vaccinated.^42^ Importantly, many of these risk factors and comorbidities had well-established associations with the gut microbiome.^43–51^ Through mediation analysis, a study has revealed that host factors such as biological sex, BMI, and COVID-19 vaccination may play a significant role in PACS via gut microbial species and functional pathways.^36^

While most human studies have only demonstrated the associations between gut microbiome dysbiosis and PACS, the important question is whether the microbial alterations were the cause and/or the consequence of PACS (i.e., the chicken or the egg question). Emerging animal experiments have highlighted the possible causal effects. An *in vivo* study has found that fecal microbiota transplantation (FMT) from PACS patients to germ-free mice resulted in lung inflammation in the absence of SARS-CoV-2, worse pathological changes in the lungs following multidrug-resistant *Klebsiella pneumoniae* infection, as well as cognitive impairment.^52^ Interestingly, another murine model has demonstrated that probiotic supplementation of *Bifidobacterium longum* could partially reverse memory impairment induced by coronavirus infection, implying the neuroprotective potential of microbiome-based therapeutics in treating post-infection cognitive conditions.^52^ Collectively, these initial findings have provided evidence supporting a direct causal link between gut microbiome dysbiosis associated with PACS and the development of impairments in both respiratory and non-respiratory functions.

## Microbiome-based therapeutics for PACS

Gut microbiome modulation using probiotics and prebiotics has been widely indicated in the treatment or management of various diseases, including but not limited to inflammatory bowel disease (IBD), irritable bowel syndrome (IBS), mood disorders, and even cancer.^53^ Fecal microbiota transplantation (FMT), which is the infusion of feces from healthy donors to the gut of individuals with a disease, has shown robust therapeutic effects for recurrent *Clostridioides difficile* infection and other emerging indications.^54–58^ During the pandemic, retrospective studies and clinical trials on probiotics, prebiotics, and FMT have shed light on the efficacy of gut microbiome modulation in the management of COVID-19.^59–66^ Given the robust relationships between the persistent symptoms and the gut microbiome, there is a potential to use these interventions in PACS patients.

A large-scale, randomized, double-blind, placebo-controlled trial (*n* = 463) has shown that a synbiotic preparation (SIM01) comprising three *Bifidobacterium* species and prebiotic compounds was effective and safe in alleviating multiple symptoms of PACS.^67^ PACS patients were randomized at a 1:1 ratio to receive SIM01 or placebo orally for 6 months. Compared to the placebo group, significantly higher proportions of subjects in the SIM01 group experienced alleviation in fatigue, memory loss, difficulty in concentration, gastrointestinal upset, and general unwellness. SIM01 treatment significantly modulated gut microbiome composition, increasing bacterial diversity and richness. In particular, the relative abundance of *Bifidobacterium* species, *Roseburia intestinalis*, *Roseburia hominis*, *Faecalibacterium prausnitzii*, and *Akkermansia muciniphila* was elevated, while that of *Ruminococcus gnavus* and *Klebsiella* species were reduced. After SIM01 treatment, several microbial pathways involved in short-chain fatty acid production, such as pyruvate fermentation to acetate and lactate (PWY-5100), were also enriched. Importantly, it was reported that *Bifidobacterium adolescentis* was positively correlated with the improvement in fatigue, gastrointestinal upset, and memory loss; *Bifidobacterium bifidum* was positively correlated with the improvement in fatigue and general unwellness; and *Bifidobacterium longum* was positively correlated with the improvement in concentration, although the exact underlying mechanisms are still largely unknown. With regard to safety, SIM01 was well tolerated with no statistical difference in adverse event rates between SIM01 and placebo groups. No adverse event was deemed likely to be related to SIM01 treatment as evaluated by an independent safety adjudication committee.

Coherently, a few studies have also observed clinical benefits of microbiome-based therapeutics on fatigue, neuropsychiatric, and gastrointestinal symptoms of PACS.^68–70^ A randomized, double-blind, placebo-controlled trial (*n* = 200) explored the use of probiotics consisting of three *Bacillus* species combined with systemic enzymes in patients with post-COVID fatigue.^68^ Patients were randomized at a 1:1 ratio to receive the intervention or placebo orally for 14 days. Significantly higher proportion of subjects had resolution of fatigue in the intervention group on day 14. The intervention group has also achieved a significantly greater improvement in the total, physical, and mental fatigue scores on the Chalder Fatigue scale (CFQ-11), without adverse events reported. A small-scale pilot study in PACS patients (*n* = 6) has demonstrated the potential of “paraprobiotics” (i.e., non-viable probiotics) in alleviating neuropsychiatric symptoms of PACS.^69^ Six patients were given “paraprobiotics” comprising 30 *Bifidobacterium*, *Lactobacillus*, *Streptococcus*, and *Saccharomyces* species for 4 weeks, followed by symptom assessments using questionnaires, mobile applications and wearable sensors. Positive effects were seen in questionnaire scores related to dysautonomia, fatigue, and depression, as well as daily steps, fatigue, and sleep quality data collected from mobile application and wearable sensors. Reductions in the expression of activation markers on several immune cells and the expression of toll-like receptor 2 (TLR2) on T cells were observed following treatment. In addition, a case report on a patient with gastrointestinal symptoms of PACS has shown that a high-fiber nutritional intervention (NBT-NM108) was associated with alleviations in loss of appetite, nausea, and anxiety.^70^ Gut microbial alterations related to symptom alleviation were observed, including an increase in potential short-chain fatty acid producers.

However, not all microbiome-based interventions were able to exert positive effects on PACS symptoms. A randomized, double-blind, placebo-controlled trial (*n* = 52) has assessed the effects of oral supplementation of a synbiotic formula containing *Bifidobacterium* and *Lactobacillus* species, prebiotic compounds, quercetin, calcium butyrate, or placebo (1:1 ratio) for 35 days starting from COVID-19 diagnosis.^71^ At 6 months, no significant difference in post-COVID symptoms was observed between the synbiotic and placebo groups. Nonetheless, the intervention prevented the decrease in microbial diversity (measured by Shannon index) during the acute phase of COVID-19, which was observed in the placebo group on days 14 and 28. Another randomized, double-blind, placebo-controlled trial (*n* = 73) has shown that a 28-day probiotic intervention with *Bifidobacterium* and *Lactobacillus* species did not lead to significant improvement in post-COVID symptoms at 3 months when compared to placebo, although the intervention was linked to reduced duration and severity of acute COVID-19 symptoms.^72^ Future studies are needed to elucidate the reasons and mechanisms underlying the response to microbiome-based therapeutics.

Antibiotics were commonly prescribed during the acute phase of COVID-19, especially in the early pandemic.^73^ A few small-scale retrospective studies had conflicting conclusions regarding the effects of antibiotics used during acute COVID-19 on the development of PACS.^74–76^ While a retrospective cohort study (*n* = 211) has demonstrated that early antibiotic treatment was associated with shorter duration to symptom resolution,^74^ another study (*n* = 311) has identified antibiotic treatment as a risk factor for PACS.^75^ To date, no prospective clinical trial has been done to evaluate the effects of antibiotics on PACS symptoms. A significant downside to the use of antibiotics is the development of antibiotic resistance.^77^ Longitudinal gut microbiome analysis in COVID-19 patients (*n* = 142) has shown that antibiotic treatment was linked to an increased abundance of antimicrobial resistance genes (ARGs).^78^ Interestingly, ARGs were found to be significantly higher in PACS patients when compared to healthy controls.^78^ Yet, the role of antibiotics in the pathogenesis of PACS is still unknown.

Last but not least, an alternative way to modulate the gut microbiome is fecal microbiota transplantation (FMT). A non-randomized, open-label prospective interventional study (*n* = 60) demonstrated that FMT is effective and safe in alleviating post-COVID insomnia and anxiety, improving sleep quality, and reducing daytime sleepiness in PACS patients.^79^ In this study, PACS patients with insomnia were allocated to receive FMT via oesophago-gastro-duodenoscopy (OGD) and flexible sigmoidoscopy (FS) or no treatment at a 1:1 ratio. At 12 weeks, a significantly higher proportion of subjects in the FMT group achieved insomnia remission when compared to the control group. Insomnia Severity Index (ISI), Pittsburgh Sleep Quality Index (PSQI), Generalized Anxiety Disorder-7 (GAD-7), Epworth Sleepiness Scale (ESS) of the FMT group were significantly improved at 12 weeks, whilst no significant change was observed in the control group. Metagenomic analyses have shown a significantly increased relative abundance of *Gemmiger formicilis* and *Coprococcus comes*, as well as an elevated bacterial richness after FMT. Microbial pathways capable of producing menaquinol derivatives, such as menaquinol-7 (MK7, vitamin K2), were also significantly decreased after FMT. Moreover, a case report (*n* = 11) has also provided preliminary data on the use of FMT via oral capsule administration (4 days; 10 capsules daily) in relieving post-COVID gastrointestinal symptoms (e.g., constipation, diarrhea, abdominal pain, epigastric pain, acid reflux), fatigue, mood disturbance (e.g., anxiety, depression), and sleep disturbance.^64^ The relative abundance of the *Bifidobacterium* and *Faecalibacterium* genera was significantly enhanced following FMT. Overall, these findings provide novel insights for the treatment of PACS by modulating the gut microbiome, and future clinical trials are warranted to confirm the findings. Further multi-omic analyses will also be crucial to further uncover the host–microbe interactions following these microbiome-based interventions and to clarify the therapeutic mechanisms underlying the observed clinical benefits.

**Figure 2.**
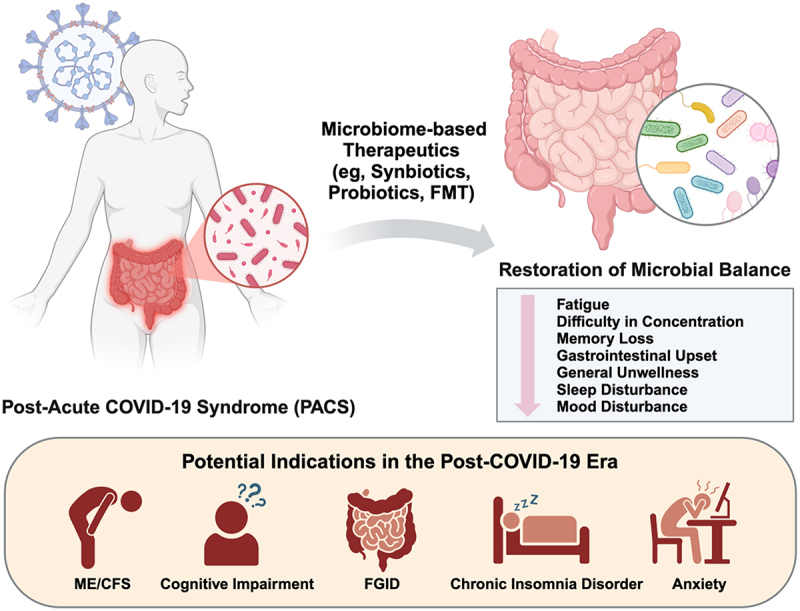
Current evidence on the use of microbiome-based therapeutics in post-acute coronavirus disease-2019 syndrome (PACS) and potential indications in the future. Recent clinical trials have indicated that gut microbiome modulation using probiotics, prebiotics, and fecal microbiota transplantation (FMT) led to improvement in multiple symptoms related to PACS, including fatigue, memory loss, difficulty in concentration, gastrointestinal upset, and disturbances in sleep and mood.^64,67–70,79^ In the future, the indications could potentially be extended to the associated chronic conditions, for instance myalgic encephalomyelitis/chronic fatigue syndrome (ME/CFS), cognitive impairment, functional gastrointestinal disorders (FGID), chronic insomnia disorder and generalized anxiety disorder. Large-scale clinical trials are warranted to confirm the efficacy and safety. COVID-19, coronavirus disease 2019; PACS, post-acute COVID-19 syndrome; FMT, fecal microbiota transplantation; ME/CFS, myalgic encephalomyelitis/chronic fatigue syndrome; FGID, functional gastrointestinal disorders. Figure created with BioRender.com.

Currently, effective treatment for PACS is limited. Several pharmacological and non-pharmacological options, including the use of antiviral or anti-diabetic drugs, nutritional supplementation, or traditional medicine, have shown conflicting results for the treatment and prevention of PACS and are still in their infancy.^70,80–88^ Although COVID-19 vaccination was shown to have protective effects against PACS in multiple studies,^89–92^ these observations could be variable based on the vaccines being used or investigated. In addition, existing literature has only demonstrated the prophylactic benefits but not the therapeutic effects of COVID-19 vaccination on PACS. Thus, alternative treatment options are needed to manage the symptoms in PACS patients. Moving forward, future large-scale clinical trials using microbiome-based therapeutics could focus on the long-term symptoms that were significantly alleviated in the previous studies, including fatigue, gastrointestinal upset, difficulty in concentration, memory loss, insomnia, and anxiety. Beyond PACS, the indications could potentially be extended to the associated chronic conditions, for instance myalgic encephalomyelitis/chronic fatigue syndrome (ME/CFS), cognitive impairment, functional gastrointestinal disorders (FGID), chronic insomnia disorder, and generalized anxiety disorder (Figure 2). Validated and objective tools could be adopted in the assessment. In the pilot study on FMT, both the upper and lower routes were used for FMT administration to target the dysbiosis in the small and large bowel, although the small bowel microbiome is largely unexplored in human studies to date. To discern the potential impact of FMT administration via the upper route, multiple arms involving small bowel alone, large bowel alone, as well as small and large bowel in combination versus control could be incorporated in future trials.

## Conclusion

The contribution of gut microbiome dysbiosis in post-acute coronavirus disease 2019 syndrome (PACS) has been widely reported across studies globally. While observational studies have identified key microbial alterations and provided important insights into the role of gut dysbiosis in the pathogenesis of PACS, emerging clinical trials have demonstrated robust effects of gut microbiome modulation in the management of acute and post-acute symptoms. In the future, mechanistic studies will be important to further elucidate the plausible mechanisms to account for the observed clinical benefits. Collectively, these data could potentially drive microbiome discoveries toward clinical applications to combat emerging infections and other chronic conditions.

## Data Availability

Data sharing is not applicable to this review article.
